# A Case Report of Squamous Cell Carcinoma Misdiagnosed as Cryptogenic Organizing Pneumonia

**DOI:** 10.7759/cureus.42574

**Published:** 2023-07-27

**Authors:** Krishna Chaudhary, Preetraj Kaur, Binod Poudel, Kyle Schroeder, Vinod Khatri

**Affiliations:** 1 Internal Medicine, Mercy Health St. Vincent Medical Center, Toledo, USA; 2 Internal Medicine, Jefferson Abington Hospital, Abington, USA

**Keywords:** pet scans, immunohistochemistry, lung biopsy, organizing pneumonia, squamous cell lung cancer

## Abstract

After adenocarcinoma, squamous cell lung cancer is the most common type of non-small cell lung cancer (NSCLC) among non-smokers. A tissue biopsy followed by imaging (chest X-ray, computed tomography (CT) lung, and positron emission tomography (PET) scan) is the best modality for confirmation and staging of the disease. Sometimes, the histopathological appearance of squamous cell lung carcinoma (SCLC) can be confused with organizing pneumonia. Such findings can delay the diagnosis of SCLC, which can affect the treatment and course of the disease. Any lung mass that is highly suspicious for carcinoma should be closely monitored with imaging, and a repeat tissue biopsy should be done for a confirmation of the diagnosis to start appropriate therapy as soon as possible.

## Introduction

Cryptogenic organizing pneumonia (COP), formerly known as bronchiolitis obliterans organizing pneumonia (BOOP), occurs secondary to injury to the alveolar wall and typically presents in the fifth or sixth decade of life, affecting both males and females in equal proportions [1]. Interestingly, COP may mimic several different disease pathologies, including but not limited to community-acquired pneumonia, idiopathic interstitial pneumonia (IIPs), and lung carcinoma [1-3]. Similarly, lung carcinoma can mimic COP both clinically and histopathologically [4]. The presenting features of COP may remain non-specific and often overlap with lung carcinoma [1,5]. The usual symptoms may range from nonproductive cough, mild fever to malaise, anorexia, weight loss, and progressive dyspnea [1,4]. Although not commonly reported, organizing pneumonia may be a manifestation of lung cancer and cause lung cancer-associated organizing pneumonia (OP) [6]. Although it is a rare manifestation, it is usually seen post-chemotherapy or radiation [6].

## Case presentation

**Figure 1:**
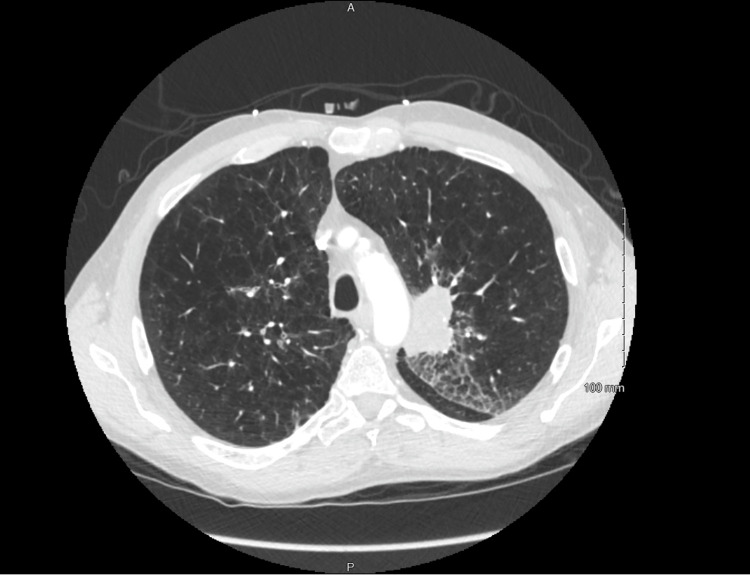
5 cm x 3 cm lobulated left supra-hilar mass abutting the aortic arch, likely malignant with areas of necrosis and mediastinal and hilar lymph adenopathy

**Figure 2:**
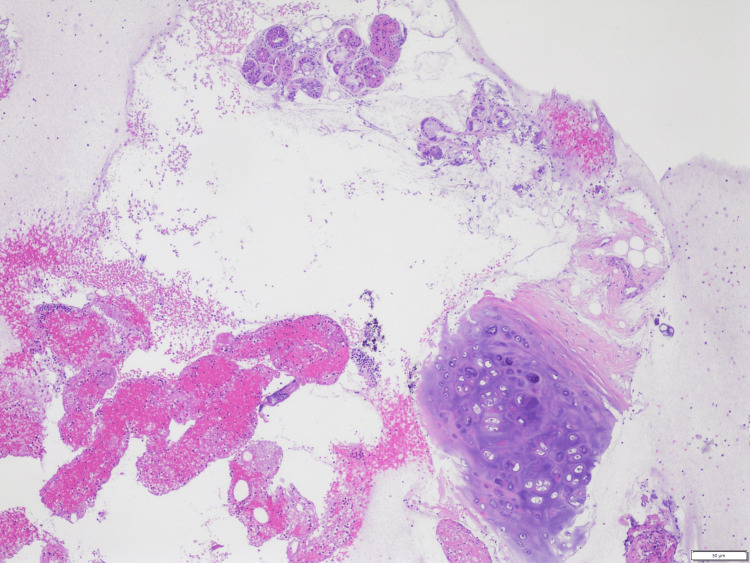
H&E stained section of mediastinal LN showing few inflammatory cells with a small piece of benign squamous epithelium and a few benign respiratory columnar cells

A 65-year-old Caucasian male with a past medical history of chronic obstructive pulmonary disorder (COPD) stage III and chronic tobacco abuse (20 packs per year) presented with worsening shortness of breath and cough. Bilateral decreased breath sounds with few rhonchi and wheezing were noted on chest auscultation. Complete blood count showed leukocytosis with a white blood cell of 21 k/uL and normocytic anemia with hemoglobin of 10 g/dl. Sputum culture showed normal respiratory flora. The respiratory viral panel was negative. CT chest revealed a 5 cm x 3 cm lobulated left supra-hilar mass abutting the aortic arch, with areas of necrosis and mediastinal and hilar lymphadenopathy (Figure 1). He was known to have mediastinal lymphadenopathy for two years, with inconsistent follow-up. He had previously undergone endobronchial ultrasound (EBUS) with transbronchial needle aspiration (TBNA) of mediastinal LN without any evidence of malignancy in the surgical pathology report (Figure 2). He was admitted and treated with IV antibiotics, bronchodilators, and methylprednisolone, with some improvement in his symptoms. Bronchoscopy was done and tissue samples were obtained from the left upper lobe and multiple mediastinal lymph nodes. The histopathology report was grossly unremarkable.

**Figure 3:**
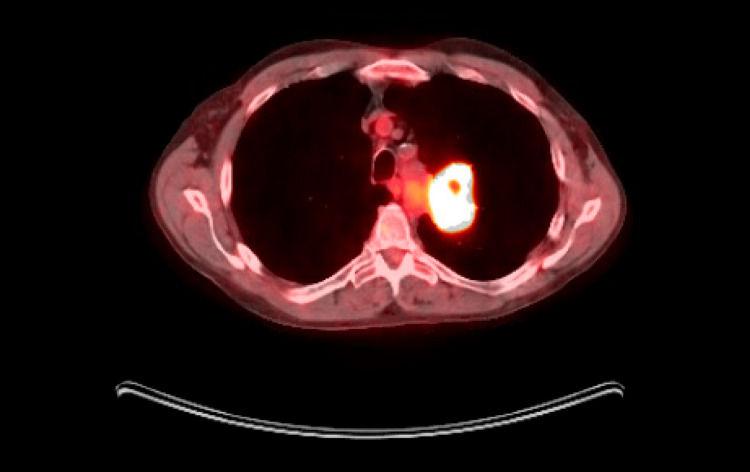
Severe FDG avidity involving a 5 x 3 cm left upper lobe mass in the supra-hilar area abutting the mediastinum, consistent with neoplasia FDG: fluorodeoxyglucose

**Figure 4:**
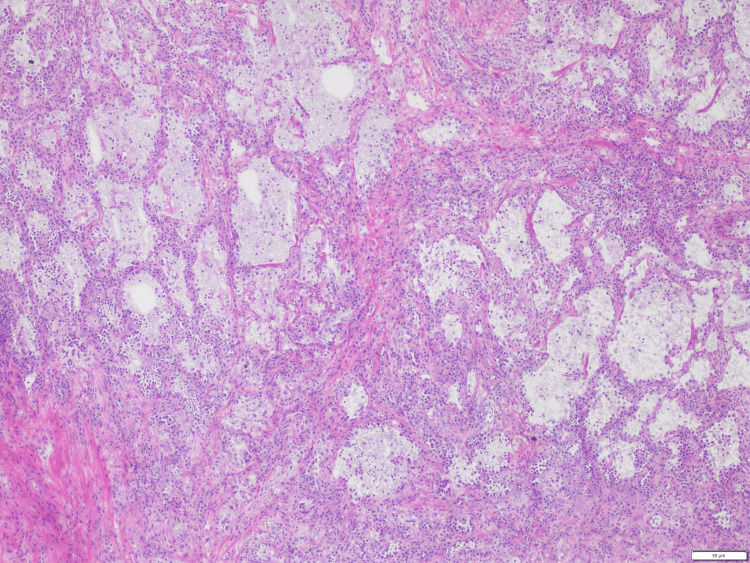
H&E stained section of lung tissue showing reactive pneumocytes and monocytes associated with organizing pneumonia H&E: hematoxylin and eosin

He was discharged on a short course of oral antibiotics, bronchodilators, and prednisone along with recommendations for an outpatient positron emission tomography (PET) scan. The PET scan showed severe fluorodeoxyglucose (FDG) avidity involving a 5 x 3 cm left upper lobe mass in the supra-hilar area abutting the mediastinum consistent with neoplasia (Figure 3). No other suspicious areas of radiotracer uptake were noted. Further plan of care was discussed with the patient for possible CT-guided biopsy or video-assisted thoracic surgery (VATS) lung biopsy. As he had undergone multiple EBUS-TBNAs previously without definitive results, he showed an interest in VATS lung biopsy for the ultimate diagnosis. During the VATS procedure, the left upper lobe lung mass of 5 cm X 3 cm was identified, which was noted to be firm and rubbery. VATS followed by wedge resection of the left upper lobe mass was done, which was negative for malignancy in the pathological report; however, the features were suggestive of inactive and organizing pneumonia (Figure 4). The patient was prescribed a short tapering course of steroids and recommended to maintain close follow-up with pulmonology.

**Figure 5:**
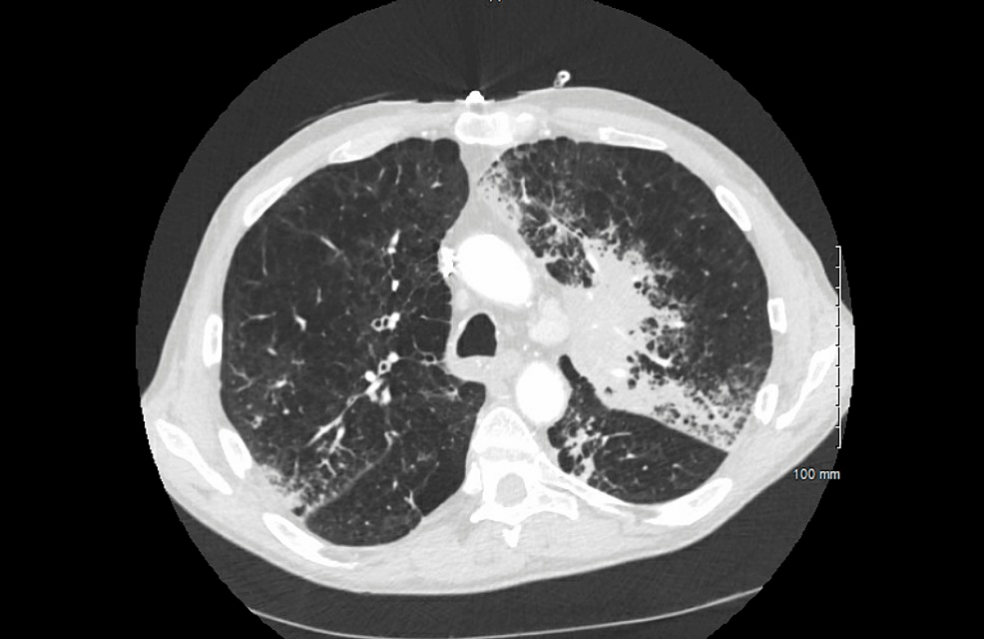
Enlargement of the left upper lung supra-hilar mass measuring 11 cm x 5 cm, with enlarged mediastinal lymphadenopathy

A month later, he was again hospitalized with complaints of worsening cough and shortness of breath. A repeat CT scan of the chest showed an enlarged left supra-hilar mass (now measuring 11 x 5 cm), enlarged mediastinal lymph nodes, along with bilateral multifocal airspace disease (Figure 5). His symptoms improved with steroids, bronchodilators, and antibiotics. His symptoms were presumed to be related to COP based on a previous lung biopsy. He was discharged on a prolonged course of steroids along with recommendations for close outpatient pulmonology follow-up.

**Figure 6:**
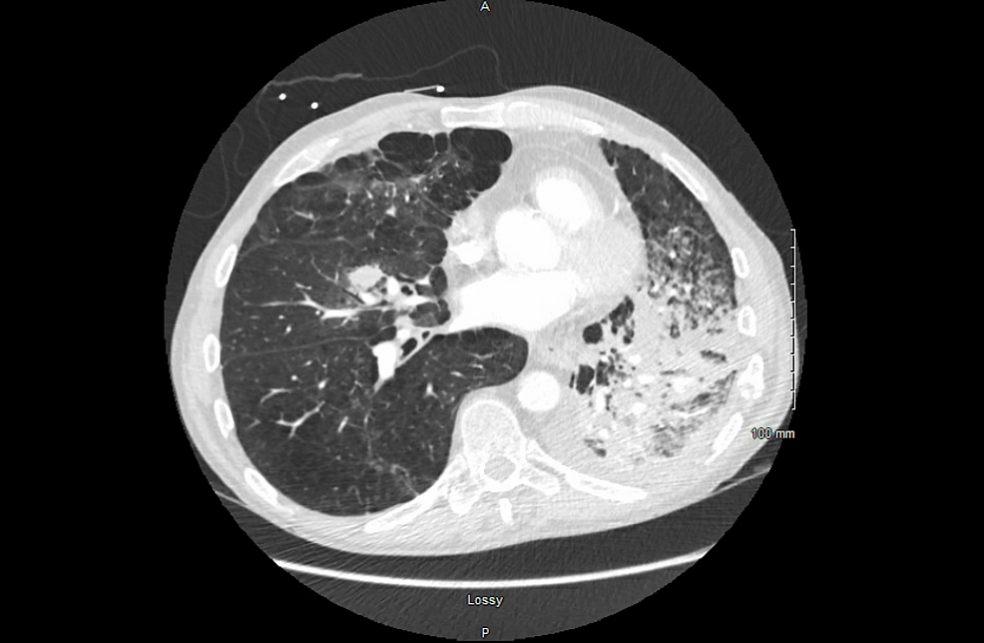
Enlargement of the left upper lung mass measuring 13 cm X 7 cm with interval development of multiple spiculated nodules throughout the right lung concerning for metastatic disease

**Figure 7:**
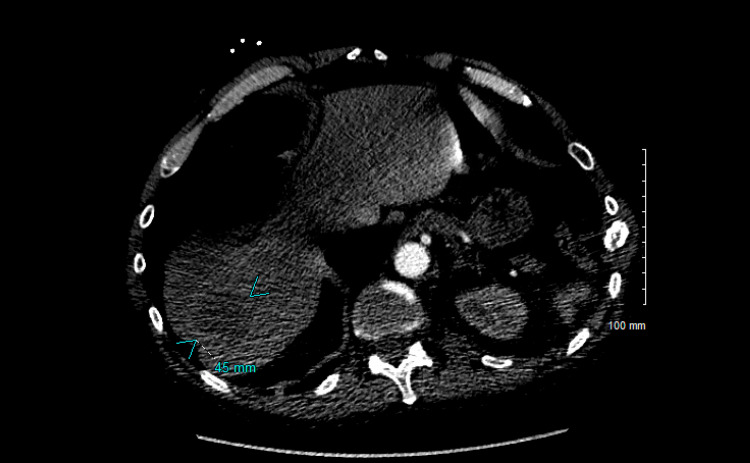
Interval development of a 4.5 cm hypodense lesion within the right lobe of the liver concerning for metastatic disease

**Figure 8:**
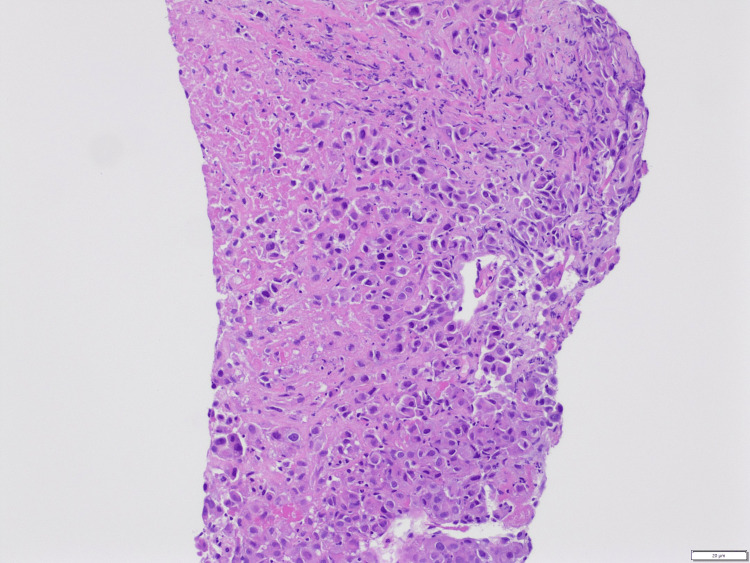
H&E stained section of liver tissue showing necrotic neoplasm with areas of viable cells; malignant cells have moderate-sized nuclei with hyperchromatic to open chromatin with some nucleoli suggestive of squamous cell carcinoma

**Figure 9:**
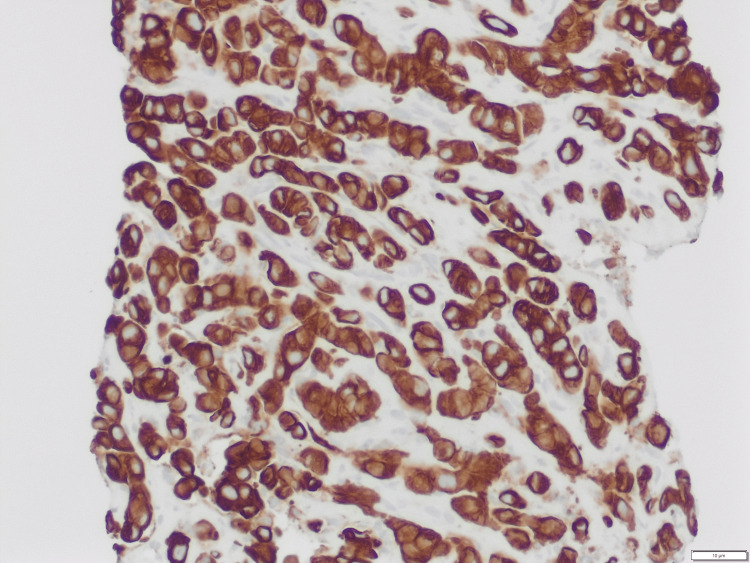
Malignant cells positive for cytokeratin 7 immunostain and patchy positive for p40 immunostain

His symptoms continued to wax and wane and thus, a repeat CT chest was done four months later, which showed progression of the left supra-hilar mass along with the development of post-obstructive pneumonia, interval development of multiple spiculated nodules throughout the right lung concerning for metastatic disease (Figure 6), and interval development of a 4.5 cm hypodense lesion within the right lobe of the liver, concerning for metastatic disease (Figure 7). A core needle biopsy of the liver mass was done, which was suggestive of squamous cell carcinoma of the lung (Figure 8). These features were supported by immunostaining (Figure 9). Oncology was consulted and the patient was eventually started on chemotherapy.

## Discussion

Squamous cell lung cancer (SCLC) is a type of non-small lung cancer that results from the transformation of squamous cells in the airway lining commonly occurring in the central part of the lung or the main airway such as the right main or left main bronchus. Chest X-ray or CT scan of the chest are initial investigation modalities following a detailed history and physical examination. Staging workup, such as a PET scan, should precede the tissue biopsy to spot any area of metastasis in highly suspicious lung lesions. It can locate an area more accessible and easier for a biopsy, confirming the diagnosis and staging at the same time. EBUS-NA (endoscopic bronchial ultrasound with needle aspiration) or EUS-NA (esophageal ultrasound with needle aspiration) can be the first approach in the patient requiring mediastinal node evaluation based on the location of the mediastinal node involved. A false negative rate is as high as 20% and highly operator-dependent [7]. Inadequate samples, internal necrosis of lymph nodes, and rare cancer types are the reason for such a high false-negative rate [8]. Tissue lung biopsy is the gold standard procedure for confirmation of the diagnosis of lung cancer [9]. CT-guided transthoracic core needle lung biopsy and video-assisted thoracic surgery for lung lesions have approximately 9% [10] and 2% [7] false-negative rates, respectively. Some cases of malignant lung lesions have been misdiagnosed as worsening sarcoidosis [11] and inflammatory myofibroblastic tumors [12] on tissue biopsy. One case of organizing pneumonia secondary to lung cancer of an unknown primary site has been reported where TBNA of 4R mediastinal lymph nodes (LN) confirmed the diagnosis of poorly differentiated sarcomatoid carcinoma arising from the alveolar epithelium [6].

Cryptogenic organizing pneumonia (COP), previously known as bronchiolitis obliterans organizing pneumonia (BOOP), is believed to be due to alveolar injury leading to the formation of granulation tissue obstructing the alveolar lumen and bronchioles, leading to respiratory failure. The exact etiology of organizing pneumonia is unknown but 31-44 % of cases have been found to be associated with a viral infection, medication, post-thoracic radiotherapy, connective tissue disorder, and cancer [13]. Cryptogenic organizing pneumonia in tissue biopsy appears as an intra-alveolar bud of granulation tissue with myofibroblast, fibroblast, and connective tissues, unlike lung squamous cell carcinoma, which is evident as polygonal cells with intercellular bridges, eosinophilic cytoplasm, and keratin pearls [14,15]. The histopathological appearance of SCLC can be mistaken for COP due to poor biopsy yield (necrosis or operator error). We should reconsider our differential diagnosis when COP doesn’t respond to glucocorticoid therapy or has mediastinal/hilar lymphadenopathy [6].

In our case, the lymph node biopsy was grossly unremarkable. Lung biopsy was negative for malignancy; however, it was suggestive of COP. Luckily, the patient was closely monitored because of the high suspicion of lung malignancy due to the finding in the PET scan and the non-responsiveness of the lung mass to prednisone. The lung mass was increasing in size despite prednisone therapy, and new liver masses were noted. Later, it was decided to do a biopsy of the liver mass, which confirmed our diagnosis of metastatic squamous cell carcinoma.

## Conclusions

Squamous cell lung cancer can be incorrectly diagnosed as cryptogenic organizing pneumonia (COP) on lung biopsy just as in the case discussed above. COP is treated with long-term oral steroids and usually shows improvement in symptoms and imaging findings within a few weeks of starting treatment. If COP is unresponsive to steroid therapy, the patient should be closely followed up with repeat imaging to avoid further delay in the diagnosis of malignancy. In our case, there was an increase in the size of the lung consolidation and new lesions were noted in the right lung and liver, strongly favoring malignant etiology. The lesions were subsequently biopsied and confirmed the diagnosis of metastatic squamous cell lung cancer. Treatment for the cancer was then promptly initiated.
